# Functional Cereal Products in the Diet for Type 2 Diabetes Patients

**DOI:** 10.1155/2019/4012450

**Published:** 2019-10-21

**Authors:** Ada Krawęcka, Aldona Sobota, Emilia Sykut-Domańska

**Affiliations:** Department of Plant Food Technology and Gastronomy, Division of Engineering and Cereals Technology, University of Life Sciences, Lublin, Poland

## Abstract

Type 2 diabetes has become one of the major health problems of the modern world. It is assumed that environmental factors have a significant impact on the development of the disease, and great importance is ascribed to the diet, which can be modified accordingly. The diet can exert prophylactic and therapeutic effects; changes in the diet in advanced disease can improve the quality of life of diabetic patients and minimise the risk of complications, which are the direct cause of diabetes-related death. Functional food, which has a potentially health-enhancing effect in addition to its nutritional value, has been increasingly recognised and required. Cereal products are crucial in diabetic nutrition. Their function can additionally be enhanced by fortification with compounds with proven hypoglycaemic effects. Pasta has a low glycaemic index and is a good carrier of fortifying substances; hence, it can be highly recommended in diets for diabetic patients.

## 1. Introduction

The fight against diabetes, referred to as the disease of the 21^st^ century, is a challenge for modern generations due to its growing incidence. As shown by the World Health Organization (WHO) data, there were 108 million adults suffering from diabetes in 1980 and 422 million in 2014 [1]. The nearly fourfold increase in the incidence of the disease over several decades is an alarming signal indicating the need of implementation of intensified preventive measures. It is estimated that the number of diabetic patients will have increased to 642 million by 2040 [2]. Type 2 diabetes accounts for 90%–95% of all cases of the disease. Once a disease of the elderly, it currently affects increasing numbers of children and young adults. It is often combined with insulin resistance inducing progressive loss of insulin secretion by pancreatic beta cells. Relative insulin deficiency is manifested by elevated blood glucose levels or hyperglycaemia [3].

It is assumed that environmental factors and diet have crucial importance in the development of type 2 diabetes. It is also emphasised that there must be a primary predisposition for damage to pancreatic beta cells, i.e., the genetic background [4]. Obesity is a key risk factor; hence, excessive body weight should be reduced. In the milieu, the term “diabesity” has been coined to describe the coexistence of obesity and type 2 diabetes as elements of a mutually propelling mechanism [5]. It is also speculated that type 2 diabetes may be reversible when negative energy balance is reached [6]. Obviously, even a small body weight loss is reflected in improved glycaemia, blood pressure, and lipidogram values [7]. Yet, there is no universal diet for subjects with diabetes. A well-balanced diet has to be based on consideration of comorbidities, physical activity, and the presence of overweight or obesity in the patient [8].

## 2. Optimal Amounts of Carbohydrates in the Diabetic Diet

There is still controversy over the recommended carbohydrate intake. Many studies indicate that limitation of the intake of these components (low carbohydrate diet LCD to 26% of carbohydrates in the daily energy balance) may result in improved glucose metabolism and enhanced insulin sensitivity [9]. The results of the meta-analysis carried out by Meng et al. [10] have demonstrated a significant decline in the level of glycated haemoglobin (HbA1c) induced by the LCD diet. Other researchers have even suggested that replacement of some saturated fatty acids with carbohydrates can be associated with cardiovascular events. Further studies proved, however, the importance of the quality of carbohydrates. They demonstrated that lower consumption of energy from saturated fatty acids in favour of carbohydrates with a low glycaemic index was associated with a lower risk of myocardial infarction [11, 12]. A direct impact on the value of postprandial glucose is exerted by not only the amount but also the quality of carbohydrates in the meal [13]. The American Diabetes Association (ADA) [3] does not propose recommendations for specific intake of carbohydrates. Yet, the ADA emphasises that the intake of refined carbohydrates and added sugars should be reduced. The European Association for the Study of Diabetes (EASD) maintains that the amount of individual macroelements should be considered on an individual basis [14]. The intake of proteins and fats, which reduce the dynamics of sugar digestion and contribute to low postprandial glucose and normal carbohydrate metabolism, seems to be important as well [15]. The debate on the quality of carbohydrates should not ignore the importance of the glycaemic index (GI), which is an indicator of the dynamics of hydrolysis and absorption of carbohydrates into the bloodstream. The glycaemic load (GL) is the value that estimates the impact of carbohydrate consumption using the glycaemic index while taking into account the amount of carbohydrates that are consumed in a serving. The aim of a diabetes diet is to provide an appropriate nutrient composition and food processing modes that will yield a meal with a low or moderate GI and GL values [16]. Carbohydrates contained in products with a low glycaemic index (GI < 50) should be the main source of energy in the diet of diabetics. It is recommended that they provide 40%–50% of the daily energy value [17]. Products with low GI contain carbohydrates that are slowly hydrolysed and gradually absorbed into the bloodstream, which allows control of postprandial glucose and insulin release [15]. The view shared by the scientists who took part in the International Scientific Consensus Summit from the International Carbohydrate Quality Consortium (ICQC, Italy, 2013) is unambiguous. The specialists agreed that a diet based on low-glycaemic-index carbohydrates is an important tool for prevention and treatment of not only diabetes but also coronary heart disease and, possibly, obesity [16]. The relationship between reduction of the intake of high GI products and prevention of type 2 diabetes is highly supported by meta-analyses of many studies, including prospective ones [18–20]. High postprandial glycaemia resulting from consumption of high GI products is regarded as one of the most important risk factors for type 2 diabetes [21].

## 3. Determinants of the Glycaemic Index in Cereal Products

### 3.1. Dietary Fibre Content

Fibre is a group of compounds comprising plant polysaccharides, oligosaccharides, lignins, fructans, and *β*-glucans, i.e., ingredients that are not decomposed by endogenous enzymes of the human gastrointestinal tract [22]. Hence, fibre does not increase the level of glycaemia, which is the first of the advantages of its presence in the diet. It is fermented by bacteria present in the colon. This process is accompanied by acidification of the colon and results in production of short-chain fatty acids (SCFA) such as acetic, propionic, and butyric acids. Therefore, cereal fibre, and especially the soluble fibre fraction, can be assigned a prebiotic effect contributing to modulation of the microbiome [23]. This results in reduction of diabetes-associated inflammation and improvement of glucose tolerance. Additionally, by preventing constipation, insoluble fibre is a prophylactic agent preventing colon cancer [24]. In turn, soluble components, i.e. *β*-glucans, pectins, and gums, increase satiety and inhibit cholesterol and glucose absorption [25, 26]. Cereal grains are rich mainly in the insoluble fraction of dietary fibre, although barley and oat products have high amounts of soluble fractions [27]. The greatest amounts of fibre are found in whole-grain milled products. Higher consumption of such products is associated with reduction of the type 2 diabetes risk in subjects with glucose intolerance [28] and decreased overall mortality among healthy population [29]. Cereal products are also a source of vitamin B, minerals, and antioxidants [27]. Excess fibre, however, can reduce absorption of the components. Therefore, the diet should be composed reasonably.

### 3.2. Content and Type of Assimilable Carbohydrates

Starch with a highly variable decomposition rate is the main carbohydrate in cereal grains. Starch is digested by endogenous glucose amylolytic enzymes. The metabolism of this monosaccharide is of particular importance in terms of obesity and diabetes. The released glucose is absorbed into the bloodstream and transported through the portal vein to the liver. In the liver, glucose is converted to glucose-6-phosphate and glycogen. The way your body uses glucose depends on its supply. If the supply of glucose is high, it is converted into glycogen. This spare polysaccharide is used by the liver when blood glucose is low or begins to fall. Insulin and glucagon are hormones that regulate blood glucose levels. Insulin lowers blood glucose; glucagon increases glucose levels at the time of hypoglycemia. It should be emphasized that the increase in insulin levels increases the active transport of glucose to fat and muscle cells [30]. The starch molecule consists of two glucose polymers: amylose and amylopectin, whose mutual ratio is very important. Amylose is an unbranched structure and, hence, more hardly accessible for amylolytic enzymes. Consequently, high-amylose starch exhibits lower digestibility and higher hydrothermal stability [31, 32]. Depending on the raw material, its content may range from <1% to 83% [33]. Starch can be divided into rapidly digestible (RDS), slowly digestible (SDS), and resistant (RS) starch (Table 1). Resistant starch is classified as a component of dietary fibre. Its presence is positively correlated with the content of amylose in the product [34, 35]. Resistant starch is not digested by endogenous enzymes of the human digestive tract but it is a valuable prebiotic that stimulates the development of bacterial flora. Aziz et al. showed that a diet rich in RS reduces weight gain, reduces fat mass and reduces the body's glycaemic response and increases insulin sensitivity. Other authors emphasize that RS not only reduces the caloric value of a meal and reduces postprandial glucose, thereby reducing the risk of obesity but also stimulates the production of short chain fatty acids (SCFA), which are formed as a result of bacterial fermentation in the large intestine. These acids, absorbed by colonocytes through the portal vein, enter the liver, where they regulate the metabolism of fatty acids and cholesterol. Propionic acid, which inhibits the synthesis of fatty acids, is of particular importance. Most SCFAs are metabolized in the liver, but some of them enter the bloodstream and regulate adipogenesis (adipose tissue growth) and release of adipocytokines (adipokines)—proteins responsible, among others, for glycaemic homeostasis and lipidemia [37, 38]. The daily dose of 15 g of resistant starch has been shown not only to increase insulin sensitivity, which is important in the prophylaxis of type 2 diabetes [39], but also to reduce cholesterol levels and contribute to body weight loss [40]. There are several types of resistant starch, e.g., physically unavailable starch contained naturally in plant cells (RS_1_), digestion-resistant granules (RS_2_), retrograded starch (RS_3_), and chemically modified starch (RS_3_) [41]. From the nutritional point of view, the most interesting type is the retrograded starch (RS_3_). The phenomenon of retrogradation occurs in hydrothermally treated gelatinised starch and consists in arrangement of amylose chains (with its branched structure, amylopectin plays a minor role) into densely packed micelles. Their structure is stabilised by hydrogen bonds. The intensity of formation of resistant starch is determined by the effect of cooling temperatures [42]. Importantly, re-heating of food does not restore the original structure in retrograded starch. Investigations have demonstrated that resistant starch-containing products have a lower glycaemic index [40, 43].

## 4. Consumption of Grain Fibre as a Key Factor Lowering the Risk of Diabetes

As rightly emphasised, fibre should be an element of the daily diet for diabetic patients [45]. However, specific recommendations for consumption thereof should be considered. The Academy of Nutrition and Dietetics (AND) recommends a daily supply of 21–25 g of fibre for women and 30–38 g for men [46]. These are small amounts, given the fact that the intake of every 7 g of fibre per day reduces the risk of diabetes (relative risk, RR 0.94) [47]. There are also no detailed recommendations for the supply of cereal-derived fibre, despite the growing number of reports on the functional effects especially of the soluble fraction of dietary fibre (SDF) contained in cereals. Numerous studies, including meta-analyses and prospective studies, indicate an inverse relationship between the intake of fibre from cereal products and the risk of type 2 diabetes [24, 48, 49]. In investigations conducted by Kuijsten [50], the daily intake of 10 g of cereal fibre was associated with a significant reduction in the risk of type 2 diabetes. Threapleton [51] has reported a RR value of 0.79 per for every 7 g of fibre. Although an increased supply of fruit and vegetable fibre and insoluble dietary fibre (IDF) is associated with reduced risk of cardiovascular disease, there is no evidence that fruit and vegetable fibre can reduce the risk of diabetes [26, 47]. The controversy over the amount of consumption of products rich in complex carbohydrates and the increased wealth status in society have contributed to undermining the importance of cereals in the diet. There is a disturbing decline in consumption of cereal products related to various dietary trends that often restrict carbohydrate intake [52]. Achievement of health benefits has become an important determinant in the choice of products. Consumers are not aware that intense reduction of carbohydrate intake can exert an opposite effect. As early as in 1977, American reports highlighted the problem of reduced carbohydrate intake and the need to increase the supply of carbohydrates [53]. The quality of carbohydrates has been unsatisfactory since the consumption of highly purified cereals devoid of beneficial fibres was propagated in. Cereals became components of highly processed products, e.g. ready-to-eat breakfast cereals, which are produced with the high-temperature extrusion (HTST) method. Concurrently, there has been an increase in the incidence of type 2 diabetes and the concomitant epidemic of overweight and obesity in the USA [54]. It is puzzling whether the limited consumption of carbohydrates, especially those present in low-processed cereal products, is a favourable trend, given the global increase in the prevalence of diabetes.

## 5. Pasta as a Functional Cereal Product

With its specific nutritional properties, pasta is a cereal product that should be used in the diet for diabetic patients. The low GI of pasta is determined by the technological parameters and the specificity of raw materials used for production. With regard to glucose as a reference product (GI = 100%), the GI value for pasta is 32%–65%, 59%–89% for white bread, and 41%–93% for ready-to-eat breakfast cereal [55]. It is believed that semolina, i.e. coarse flour obtained from *Triticum durum*, is the best raw material for production of high quality pasta [56]. Semolina contains large amounts of resistant RS_1_ starch [30]. Additionally, literature data indicate a higher amount of amylose in durum wheat starch than in the hexaploid wheat (*Triticumaestivum*) starch. Noteworthy, unbranched amylose chains exhibit greater susceptibility to retrogradation [31], which results in formation of resistant RS_3_ starch and, consequently, reduction of the GI of the meal.

Low-temperature extrusion is the most advantageous pressing technology enhancing the nutritional value of pasta [57]. The pasta pressing temperature does not exceed 50°C at the pressure of up to 13 MPa [58]. The product has a very densely packed internal structure. In freshly pressed pasta, swollen starch granules are tightly surrounded by the protein matrix. This structure, preserved in the process of pasta drying, limits the swelling of starch granules during hydrothermal treatment and impedes the access of amylolytic enzymes during digestion [59]. Many authors emphasise that this specific dense microstructure is the major determinant of the low IG in pasta. It should also be underlined that pasta made of durum semolina, characterised by high protein content, requires a longer time to be digested in the stomach, which contributes to slower passage of food content into the duodenum and slows down glucose release into blood. Undigested polypeptides impede the access of amylolytic enzymes to starch, which seems important for maintenance of low postprandial glucose levels [56]. Appropriate hydrothermal treatment and preparation of al dente pasta ensure a lower glycaemic index value, as nongelatinised starch contained in the pasta core is not digested by amylolytic enzymes [55].

## 6. Methods for Fortification of Pasta

The process of food enrichment is associated with the need to create products that can improve health and well-being when consumed on a regular basis. Pasta proves to be an ideal product, given its growing popularity and high frequency of consumption in the recent years [60]. Addition of nontraditional materials to semolina results in variable process parameters and rheological properties of pasta dough [61]. It is suggested that pasta achieves sensory acceptance when the enrichment level does not exceed 10% [62]. In practical terms, this issue is debatable and depends on individual judgement. For instance, 30% enrichment of durum wheat pasta with wheat bran was assigned similar sensory quality as a control sample of whole-grain durum wheat pasta [63].

A popular method is fortification with various fibre components, which further reduces the pasta GI value. While the enrichment of pasta in cereal bran does not always give good results due to deterioration of colour, texture and increase in dry matter loss during cooking of products, good results have been obtained using the addition of RS. Research Aravind et al. [64] prove that resistant starch (RS_2_ and RS_3_) introduced into products at a level of up to 20% does not significantly affect the loss of dry matter during cooking, texture, and sensory properties of pasta, with only a minimal reduction in yellowness of the products. In addition, studies have shown that the addition of RS significantly reduces the degree of starch hydrolysis in vitro compared to the control. According to the authors, resistant starch may be ideal sources for the preparation of pasta with reduced starch digestibility. Soluble fibre fractions are mostly recommended, as many studies indicate that insoluble dietary fibre fractions do not play a significant role in determination of the GI of products. Pasta can be fortified e.g. with inulin, *β*-glucans, or guar gum [65]. Soluble fibre fractions together with the protein matrix, which is responsible for formation of the structure of products, surround starch granules, thus inhibiting digestion thereof and limiting the rate of absorption of released glucose [27]. Fortifying components such as inulin or guar gum may additionally improve the texture and colour of the product [66]. The addition of fermented materials may improve the nutritional value. Semolina pasta fortified with faba bean flour fermented with *Lactobacillus plantarum* DPPMAB24W (substitution level of 30%) increased resistant starch content [67]. Fortification of durum semolina pasta with quinoa flour (at a level of 20%) fermented with *Lactobacillus plantarum* T6B10 and *Lactobacillus rossiae* T0A16 increased content of fibre, protein, and antioxidants [68]. The addition of isolates and concentrates of vegetable protein can increases the protein content in the product. Sadeghi and Bhagya [69] reported an increase in protein content in pasta fortified with mustard protein isolate used at various levels (2.5%, 5%, 10%). Flax components may be interesting fortification agents as much as mineral ingredients. Flax is a carrier of lignans from the group of polyphenols. Moreover, it is a source of α-linolenic fatty acid. Both these components limit the development of diabetes-associated inflammation [70, 71]. Zinc, chromium, manganese, and selenium are important elements involved in carbohydrate metabolism [72, 73]. There are quite extensive investigations on the possibility of enriching pasta with functional ingredients. Nevertheless, it is still advisable to search for new attractive raw materials that could enhance the pro-health potential of cereal products and increase their use in the diet for patients with carbohydrate metabolism impairment.

## 7. Summary

High intake of starch carbohydrates with a low glycaemic index is associated with a greater supply of fibre and, consequently, contributes to reduction of the risk of type 2 diabetes. The glycaemic index of cereal products depends on the type of raw materials used for production and the technological process parameters. Pasta does not increase postprandial glucose levels dramatically. Additionally, it can be fortified with substances with proven hypoglycaemic activity, which increases its potential as a functional product for subjects with carbohydrate metabolism disorders, in particular for diabetes patients.

## Figures and Tables

**Table 1:** Classification of starch [44].

Type of starch	Example of occurrence	Probable digestion in small intestine
Rapidly digestible (RDS)	Freshly cooked starchy food	Rapid
Slowly digestible (SDS)	Most raw cereals	Slowly, but completely

*Resistant starch (RS)*		
RS_1_ physically inaccessible	Partly milled grains and seeds	Resistant
RS_2_ resistant granules	Raw potato, green banana, high amylose maize starch	Resistant
RS_3_ retrograded starch	Cooked and cooled starchy foods, bread and cornflakes	Resistant
RS_4_ chemically modified starch	Starch ethers, starch esters and crossbonded starches	Resistant
